# Bridging the policy–practice gap: A call for urgent early intervention and early education South African policy reform for children who are deaf or hard-of-hearing

**DOI:** 10.4102/sajcd.v73i1.1156

**Published:** 2026-08-14

**Authors:** Luisa Petrocchi-Bartal

**Affiliations:** 1 Department of Speech Pathology and Audiology, School of Human and Community Development, Faculty of Humanities, University of the Witwatersrand, Johannesburg, South Africa

**Keywords:** early intervention, early education, deaf or hard-of-hearing, policy implementation, South Africa, intersectoral collaboration, early childhood development

## Abstract

In the global context, South Africa demonstrates higher disabling-hearing-loss prevalence rates than high-income countries because of its contextual challenges such as high burden of disease and audiology service access inequity. While high-income countries demonstrate integrated Early Hearing Detection and Intervention (EHDI) programmes with mandated support, South Africa faces distinctive challenges in translating its early intervention policy precepts into actionable support systems, with fragmentation across health, social development, and education sectors for children who are deaf or hard-of-hearing (D or HH). Key barriers include delayed detection and diagnosis, reduced access to audiological services and education, cultural and linguistic incongruence, and inadequate data management systems, particularly as children who are D or HH transition between health, education and social development sectors.

**Contribution:**

Despite policy recognition of South African Sign Language and calls for inclusive education, service delivery remains siloed and reactive rather than proactive. The South African disconnect between policy intent and practice necessitates urgent reform through mandating implementation of EHDI guidelines, the integration of interdepartmental referral systems, and bottom-up legislative processes that incorporate grassroots stakeholder input. Culturally and linguistically responsive approaches and system integration, with cognisance of child and/or family culture and home language across system tiers (specifically the micro-, meso- and exo-system levels), are essential to ensure equitable developmental outcomes for children who are D or HH in South Africa. This clinical audiologist and researcher’s opinion piece offers tangible recommendations for the way forward, including mandating EHDI guidelines, developing policy briefs, using early education as a nexus for diagnosis and intervention, and fostering bottom-up grassroots stakeholder involvement.

## Introduction

Disabling hearing loss – World Health Organization (WHO, 2025) terminology referring to hearing loss in the better ear exceeding 35 dB – places children at elevated risk for compromised communicative (speech, oral and sign language), cognitive, literacy, and psychosocial development, possibly limiting long-term vocational potential (WHO, 2021, 2025). To mitigate these impacts, early intervention (EI) is recommended by the WHO (2021, 2025), the Joint Committee on Infant Hearing (JCIH) (2013, 2019), the consensus panel Family-Centred Early Intervention for Deaf and Hard-of-Hearing Children (FCEI-DHH) (Moeller et al., 2024), and the Health Professions Council of South Africa (HPCSA, 2018). All four guiding documents converge on the same core Early Hearing Detection and Intervention (EHDI) principles: Prompt identification and diagnostic confirmation followed by rapid family-centred, linguistically and culturally sensitive EI enrolment; EI is to include timely access to amplification, habilitation, counselling, multidisciplinary coordination, ongoing surveillance, and interoperable systems to ensure follow-up across health, education and social sectors (HPCSA, 2018; JCIH, 2013, 2019; Moeller et al., 2024; Szarkowski et al., 2024; WHO, 2021, 2025). The HPCSA (2018) aligns with these international tenets but explicitly adapts them to South African realities by, for example, specifying a context-specific timeline for EI, with a maximum age of 8 months.

Within the South African context, early childhood development (ECD) spans conception to school entry, typically around age 6 years, or 7 years for children with disabilities (Department of Social Development [DSD], 2015). Early intervention and early education (EE), as part of South Africa’s ECD service package include EI and EE for children who are deaf or hard-of-hearing (D or HH) (DSD, 2015) from 36 months through to Grade R (typically age 5 years turning 6 years), guided by the National Curriculum Framework for children from birth to 4 years and the Curriculum Assessment Policy Statement (CAPS) Foundation Phase inclusive of Grade R (Department of Basic Education [DBE], 2011, 2015). This period supports holistic cognitive, physical, emotional, and social development towards Grade 1 readiness (DBE, 2011, 2015). Accommodations for hearing loss occur in mainstream or special education settings and may include curriculum adjustments and sign language (DBE, 2011). Navigating early childhood hearing loss requires coordinated support across health, education, and social development divisions, especially during intersectoral transitions (HPCSA, 2018), with involvement from governmental departments at all levels of care, including health, social development, education, and the private sector (HPCSA, 2018, p. 8). However, HPCSA guidelines (2018) are non-legislative and lack clear mechanisms to operationalise inter-sectoral collaboration (HPCSA, 2018; Petrocchi-Bartal et al., 2025). Early Hearing Detection and Intervention implementation thus varies substantially across public and private domains. Systemic challenges persist where interdepartmental policy overlap between health, education, and social development and reduced interdepartmental cohesion hinder coordinated service delivery (Moodley, 2021; Petrocchi-Bartal et al., 2025).

This article adopts a systems theory lens (Bronfenbrenner, 1979; Bronfenbrenner & Morris, 2006) to reflect how nested health, education, and social development systems interact to shape outcomes for children who are D or HH in South Africa.

## The South African context: Policy intent

South Africa’s post-apartheid policy advances (the 2030 National Development Plan, Republic of South Africa, 2012), DSD’s ECD Integrated Programme of Action (DSD, 2013), and the National Integrated ECD Policy (Department of Social Development [DSD], 2015) demonstrate commitment to inclusive ECD. Statutory responsibilities are distributed across sectors – Department of Health (DoH) for early identification, rehabilitation and assistive devices (DSD, 2016), DBE for education and special education, DSD for social supports – yet the DoH, DSD, DBE interface for the transition of services remains challenging (Petrocchi-Bartal et al., 2025).

A recent South African policy review on children aged 6 years and below who are D or HH revealed largely cursory references to culturally sensitive, family-centred, multi-disciplinary, multi-sectoral considerations, with superficial and often absent D or HH-specific details (Petrocchi-Bartal et al., 2025). The DoH’s Framework and Strategy for Disability and Rehabilitation Services (DoH, 2015) emerged as the only document that explicitly references EHDI benchmarks and aligns with international standards such as the JCIH 1:3:6 timeline, but it omits reference to the HPCSA’s (2007, 2018) contextualised EHDI guidelines. The DoH’s strategic documents mention audiological services and rehabilitation, yet budgetary constraints and provincial disparities hinder execution (National Treasury, 2016; Rabothata, 2018). Provinces such as Gauteng have prioritised infrastructure for special-needs schools, but others lag behind (Tandwa, 2017).

Supportive EI and EE policy for children who are D or HH includes recognition of South African Sign Language (SASL), the provision of assistive devices such as hearing aids, and calls for inclusive education and intersectoral collaboration, but specific details of EI and EE remain scant (Petrocchi-Bartal et al., 2025). Specifically, the White Paper on the Rights of Persons with Disabilities promotes SASL training for educators and parents and acknowledges the Deaf community’s linguistic identity (DSD, 2016). Education White Paper 6 and the Screening, Identification, and Assessment, Support Policy advocate for early screening and curriculum adaptation for learners who are D or HH (Department of Education, 2001; DBE, 2014). Despite these D or HH accommodations, policy fragmentation persists with siloed service delivery and poor transitions between EI and EE settings (Petrocchi-Bartal et al., 2025). While the National Integrated ECD Policy outlines multi-sectoral roles, it fails to operationalise mechanisms for providing D- or HH-specific services (DSD, 2015). Moreover, data systems remain underdeveloped, with no national registry to track children who are D or HH across sectors (Moodley & Störbeck, 2017). Despite this robust policy landscape, execution remains uneven and fragmented.

## South African implementation challenges: From policy to practice

**FIGURE 1:**
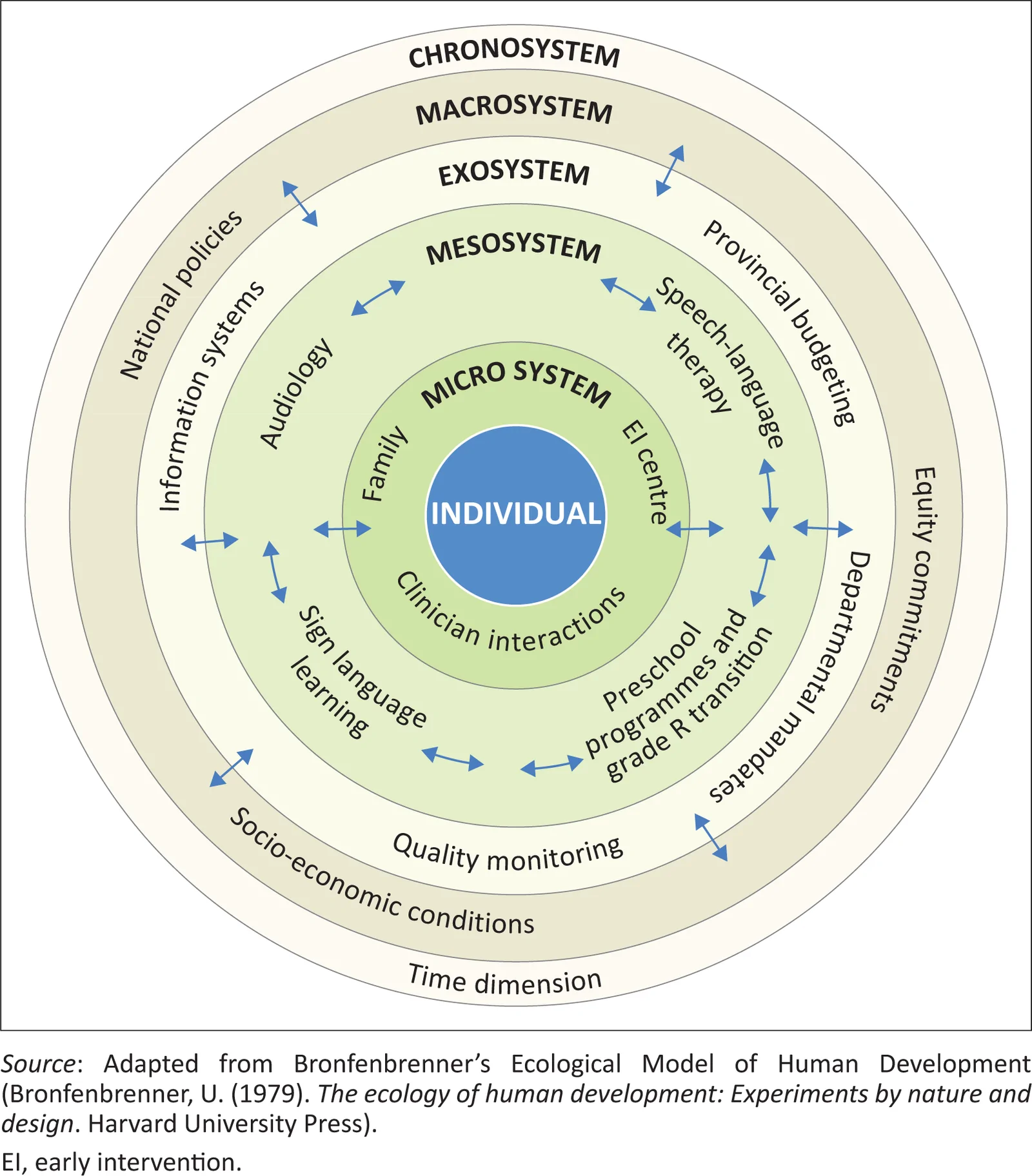
Ecological system’s perspective – Deaf or hard-of-hearing child within the South African context.

Fragmentation across health, education, and social development impedes the coordination of EI and EE for children who are D or HH, particularly in inter-sector transitions and integrated service provision (Petrocchi-Bartal et al., 2025). For the child who is D or HH, the adopted systems perspective (Bronfenbrenner, 1979; Bronfenbrenner & Morris, 2006), clarifies how deficits in one subsystem reverberate across others and are shaped by broader systemic inequities such as poverty, service provision, cultural disconnects, and linguistic exclusion (Venter, 2022). Figure 1 depicts the South African scenario regarding its nested systems that impact the individual, enabling systematic scrutiny of how policy stipulations either facilitate or fragment EI for the child who is D or HH.

Specifically, Karisa et al. (2022) highlight systemic breakdown and argue that South Africa’s ECD system, in which EI and EE are located (DSD, 2015), remains structurally exclusionary for children with disabilities. Multiple studies document these breakdowns, such as late screening and diagnosis, inconsistent follow-up and rescreening attrition, and consequent delayed audiological EI (average screening 13.5 months; mean diagnosis 32 months) (Kanji & Opperman, 2015; Khoza-Shangase, 2019; Khoza-Shangase & Michal, 2014; Maluleke et al., 2019, 2024). Caregivers report limited service availability, long travel distances, affordability barriers, and frustration with limited communication gains and breakdowns in mainstream placements (Khoza-Shangase, 2019; Khoza-Shangase & Maluleke, 2025; Maluleke et al., 2024). In addition, South Africa’s two-tiered public–private health system concentrates well-resourced EHDI services in the private sector, while the public sector, serving the majority, remains under-resourced and unevenly distributed (Naidoo & Khan, 2022; Ranchod et al., 2017). Audiology services are often delivered in English (and Afrikaans) despite most caregivers’ non-English languages at home, and SASL provision and culturally appropriate materials are inconsistent (Casoojee et al., 2024; Khoza-Shangase & Maluleke, 2025; Khoza-Shangase & Mophosho, 2018). Data management is patchy and largely paper-based, with no national registry, thereby limiting government tracking, planning and intersectoral coordination (Alam et al., 2016; Moodley & Störbeck, 2017).

Public–private systemic inequities, public-sector constraints, South Africa’s multilingual and multicultural contextual complexities, and reduced intersectoral collaboration, despite the HPCSA’s (2018) acknowledgement, require political will to drive the successful translation of policy intent into equitable policy application.

These multi-level breakdowns illustrate the systemic misalignment, viewed through a systems theory perspective, that compounds to limit EI effectiveness and EE outcomes, thereby perpetuating inequities (Hall et al., 2019).

## Towards solutions: Cultural and linguistic responsiveness and system integration

Addressing these entrenched challenges requires coordinated top-down (policy enforcement) and bottom-up (community and stakeholder inclusion) reforms. To achieve ‘bottom-up legislative reform’, approaches such as structured policy dialogues, community consultation fora, or participatory policymaking could be adopted. Störbeck (2024) posits that investing in family-centred early childhood interventions is a moral imperative and that resource allocation must be complemented by culturally grounded stakeholder engagement. Culturally and linguistically responsive approaches (SASL, oralism, bilingualism) to caregiver concerns within systems are thus advocated to close the gaps in EI audiology services and EE reported by caregivers of children who are D or HH (Casoojee et al., 2024; Maluleke et al., 2021, 2024). To this end, Casoojee et al. (2024) recommend collaborative initiatives to address systemic barriers, particularly the need to use linguistic alternatives to English and Afrikaans, as well as culturally appropriate therapy materials, for a more inclusive audiology EI approach. Similarly, Maluleke (2024) stresses the importance of aligning EHDI models with caregiver cultural values and language preferences (SASL, oralism, bilingualism), echoing the HPCSA’s family-centred framework. It must, however, be acknowledged that aligning child language preference (SASL, oralism, bilingualism) with family language preference may be challenging, given South Africa’s linguistic diversity, with 12 South African official languages recognised. Nonetheless, without mandated national policy, even impactful, free, family-centred, home-based EI programme models such as HI HOPES require government support to sustain (Störbeck, 2024). Petrocchi-Bartal et al. (2025) highlight the need for improved intersectoral communication between the Departments of Health and Basic Education to facilitate continuity of care, especially during EI-to-EE transitions.

In view of the above patent disconnect between policy intent and practice realities for EI and EE for children who are D or HH, Petrocchi-Bartal et al. (2025) recommend a bottom-up legislative reform process grounded in direct grassroots stakeholder engagement (community, caregivers, service providers). It is recommended that HPCSA guidelines be updated on this basis, including non-audiological EI pathway inclusion and detailed considerations for the intersectoral pathway for EE and social development. Thereafter, legislative application of the updated, non-audiological, and intersectorally inclusive EHDI HPCSA guidelines is recommended to ensure equitable EI and EE outcomes for children under 6 who are D or HH. Finally, establishing monitoring and evaluation systems that integrate cross-sectoral data is critical to tracking the impact of reforms and ensuring accountability.

## Conclusion

Drawing upon the identified gaps and the imperative for seamless continuity and grassroots engagement, the following are key recommendations (short-term, medium-term, and long-term) to establish clear, actionable policy navigation pathways for children who are D or HH in South Africa. They provide an extended version of the Petrocchi-Bartal et al. (2025) recommendations and collectively form a strategic blueprint for developing a responsive and ultimately more effective system of support for children who are D or HH in South Africa. Without a formalised approach at the government level, a marked portion of South African children who are D or HH will continue to be marginalised, curtailing conditions that allow for their ease of self-actualisation.

### Recommendations

Mandate comprehensive EHDI guidelines, embed intersectoral collaboration, and localise service provision with urban–rural considerations. The recommendations below are sequenced as short-, medium-, and long-term and emphasise multi-level stakeholder engagement, including caregivers and service providers.

### Short-term: Early hearing detection and intervention guidelines review

Review HPCSA EHDI guidelines (2018) to integrate EE and non-audiological pathways and to specify roles across the microsystem (family, clinician), mesosystem (preschool, therapy, sign language, Grade R transition), exosystem (information systems, provincial budgeting, departmental mandates, quality monitoring) and macrosystem (national policy, equity commitments). Design EE settings as crossover platforms for detection and early pedagogical support, given the persistent late diagnosis.

### Medium-term: Mandated early hearing detection and intervention guidelines implementation

Secure political endorsement (macro, exo, meso systems) to convert the updated HPCSA guidance into mandated national protocols with defined timelines, delineated responsibilities for DoH, DBE, and DSD, institutionalised family involvement, dedicated funding, and workforce development, prioritising underserved provinces and rural areas. Mandated rollout will mitigate late detection and improve school readiness.

### Long-term: Policy brief development for integrated interdepartmental referral systems and transitional pathways

Develop formal interdepartmental referral systems (macro, exo, meso systems) that automatically link diagnosis to EI, EE and social supports; implement centralised cross-sector data systems for tracking, monitoring and planning; conduct infrastructural audits and align budgets to operational needs; and embed intersectoral collaboration within departmental mandates to ensure continuity of support.

### Long-term: Inclusive oversight and stakeholder engagement for bottom-up system refinement

Establish a central EHDI or EE oversight committee (micro, meso, exo, macro systems) with representation from DoH, DBE, DSD, caregivers, service providers and deaf community leaders to monitor implementation, ensure cultural and linguistic congruence, and guide iterative, community-informed policy refinement.
